# Fatigue in patients with systemic lupus erythematosus and neuropsychiatric symptoms is associated with anxiety and depression rather than inflammatory disease activity

**DOI:** 10.1177/09612033211005014

**Published:** 2021-03-28

**Authors:** Rory C Monahan, Liesbeth JJ Beaart-van de Voorde, Jeroen Eikenboom, Rolf Fronczek, Margreet Kloppenburg, Huub AM Middelkoop, Gisela M Terwindt, Nic JA van der Wee, Tom WJ Huizinga, Gerda M Steup-Beekman

**Affiliations:** 1 Department of Rheumatology, Leiden University Medical Center, Leiden, the Netherlands; 2 Department of Internal Medicine, Division of Thrombosis and Hemostasis, Leiden University Medical Center, Leiden, the Netherlands; 3 Department of Neurology, Leiden University Medical Center, Leiden, the Netherlands; 4 Sleep-Wake Center SEIN, Heemstede, the Netherlands; 5 Department of Clinical Epidemiology, Leiden University Medical Center, Leiden, the Netherlands; 6 Department of Psychology, Health, Medical and Neuropsychology Unit, Leiden University, Leiden, the Netherlands; 7 Department of Psychiatry, Leiden University Medical Center, Leiden, the Netherlands; 8 Department of Rheumatology, Haaglanden Medical Center, the Hague, the Netherlands

**Keywords:** Systemic lupus erythematosus, neuropsychiatric lupus, fatigue

## Abstract

**Introduction:**

We aimed to investigate risk factors for fatigue in patients with systemic lupus erythematosus (SLE) and neuropsychiatric symptoms in order to identify potential interventional strategies.

**Methods:**

Patients visiting the neuropsychiatric SLE (NPSLE) clinic of the Leiden University Medical Center between 2007–2019 were included. In a multidisciplinary consensus meeting, SLE patients were classified as having neuropsychiatric symptoms of inflammatory origin (inflammatory phenotype) or other origin (non-inflammatory phenotype). Fatigue was assessed with the SF-36 vitality domain (VT) since 2007 and the multidimensional fatigue inventory (MFI) and visual analogue scale (VAS) since 2011. Patients with a score on the SF-36 VT ≥1 standard deviation (SD) away from the mean of age-related controls of the general population were classified as fatigued; patients ≥2 SD away were classified as extremely fatigued. Disease activity was measured using the SLE disease activity index-2000. The influence of the presence of an inflammatory phenotype, disease activity and symptoms of depression and anxiety as measured by the hospital anxiety and depression scale (HADS) was analyzed using multiple regression analyses corrected for age, sex and education.

**Results:**

348 out of 371 eligible patients filled in questionnaires and were included in this study . The majority was female (87%) and the mean age was 43 ± 14 years. 72 patients (21%) had neuropsychiatric symptoms of an inflammatory origin. Fatigue was present in 78% of all patients and extreme fatigue was present in 50% of patients with an inflammatory phenotype vs 46% in the non-inflammatory phenotype. Fatigue was similar in patients with an inflammatory phenotype compared to patients with a non-inflammatory phenotype on the SF-36 VT (β: 0.8 (95% CI −4.8; 6.1) and there was less fatigue in patients with an inflammatory phenotype on the MFI and VAS (β: −3.7 (95% CI: −6.9; −0.5) and β: −1.0 (95% CI −1.6; −0.3)). There was no association between disease activity and fatigue, but symptoms of anxiety and depression (HADS) associated strongly with all fatigue measurements.

**Conclusion:**

This study suggests that intervention strategies to target fatigue in (NP)SLE patients may need to focus on symptoms of anxiety and depression rather than immunosuppressive treatment.

## Introduction

Fatigue is reported by up to 90% of patients with systemic lupus erythematosus (SLE).^1^ Many potential underlying causes have been described, for example lack of physical activity, sleep disturbance, pain, burden of chronic disease, inflammation and disease activity.^2^

Despite the high prevalence of fatigue in patients with SLE, a clear treatment strategy is lacking. In clinical practice, patients regularly ask whether starting or increasing immunosuppressive treatment (e.g. prednisone) is beneficial. If inflammation would be an important cause of fatigue, this would be an appropriate strategy. In multiple autoimmune diseases, it has been demonstrated that the enhancement of inflammatory mediators, including interleukin (IL)-1, IL-6 and tumor necrosis factor-alpha, induces or increases fatigue.^3^ In SLE, some trials indeed have shown a potential benefit of specific anti-inflammatory therapy on fatigue. However, many patients with effectively treated SLE still suffer from residual fatigue and the association between inflammation and fatigue remains uncertain.^4,5^ Another possible explanation for fatigue is that chronic diseases increase the risk of psychiatric disorders such as anxiety and depression, which are associated with fatigue.^6^ If this is the main underlying cause, focusing on mental wellbeing with e.g. psychotherapy might be preferred.^7,8^

Up-to-date, only two studies have looked at fatigue in patients with SLE presenting with neuropsychiatric symptoms.^9,10^ Both studies demonstrated that the presence of neuropsychiatric events led to more fatigue. In addition, patients with neuropsychiatric events attributed to SLE (NPSLE) reported slightly less fatigue than patients with neuropsychiatric events due to other causes.^10^ However, in this study, no distinction was made between the currently recognized types of SLE-related events (NPSLE): of ischemic or inflammatory origin or a combination thereof.^11^ Patients with inflammatory NPSLE can have low or normal disease activity scores measured by instruments such as the Systemic Lupus Erythematosus Disease Activity Index-2000 (SLEDAI-2K),^12^ despite the presence of inflammation of the nervous system. It has also been suggested that neuroinflammation in general plays a role in the occurrence of fatigue.^13,14^ Therefore, regardless of disease activity, one would expect to find more fatigue in patients with inflammatory NPSLE.

We aimed to study the prevalence of fatigue in SLE patients with different types of neuropsychiatric involvement (inflammatory vs. non-inflammatory) and study other factors potentially associated with fatigue, in order to guide treatment strategies.

## Patients and methods

### Study design

**Figure 1.**
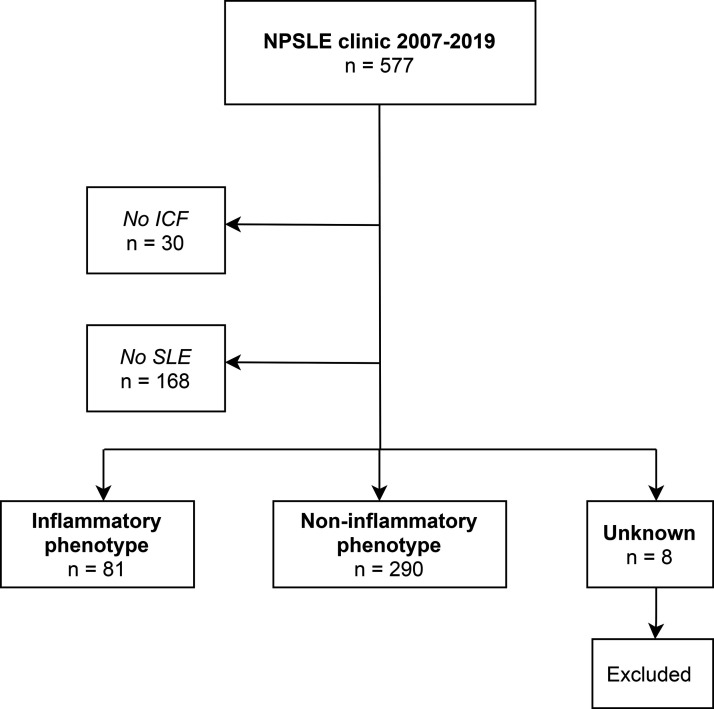
Patient inclusion. No ICF: no informed consent; SLE: systemic lupus erythematosus; NPSLE: neuropsychiatric SLE; Unknown = unclear diagnosis (NPSLE yes/no).

The NPSLE clinic of the Leiden University Medical Center (LUMC) is a tertiary referral center in the Netherlands for patients with (suspicion of) SLE and neuropsychiatric symptoms. All patients are evaluated in a multidisciplinary setting, by a rheumatologist, neurologist, clinical neuropsychologist, psychiatrist, vascular internal medicine expert and an advanced nurse practitioner, as described previously.^15^ In addition, all patients undergo brain MRI and extensive laboratory investigation. Two weeks later, a multidisciplinary meeting takes place in which a consensus is reached regarding the presence of NPSLE. Treatment advice is given to the referring physician(s) based on the presumed underlying cause of the neuropsychiatric symptoms. All consecutive patients visiting the NPSLE clinic between 2007–2019 with the diagnosis of SLE (based on clinicians’ assessment) that signed informed consent were included in the study (see Figure 1). This study was approved by the local medical ethical committee.

### Data collection

Medical records were reviewed to collect data on demographics, clinical presentation, diagnosis of SLE and NPSLE and medication use at the first visit at the NPSLE clinic of the LUMC, which took place at any time between 2007–2019. Patients were classified as NPSLE if neuropsychiatric symptoms were attributed to SLE by multidisciplinary assessment and required treatment other than symptomatic treatment. Patients with (mild) neuropsychiatric symptoms not requiring additional treatment (e.g. only anti-epileptics and not prednisone) or symptoms not attributed to SLE (e.g. alternative diagnoses) were classified as minor/non-NPSLE. For more details, we refer to previously published work.^16^ When the diagnosis of NPSLE was established, the phenotype (inflammatory, ischemic or combined) and 1999 ACR NPSLE syndromes were assigned.^17^ Patients in whom no consensus regarding the diagnosis of NPSLE was reached during multidisciplinary consensus meeting were excluded. NPSLE phenotype was based on serological and radiological evaluation and clinical judgement. For this study, patients were divided in neuropsychiatric symptoms of inflammatory origin (inflammatory or combined NPSLE) and non-inflammatory origin (ischemic or minor/non-NPSLE). Disease activity was calculated using the SLEDAI-2K, (range 0–105).^12^

#### Symptoms of anxiety and depression

Symptoms of anxiety and depression were assessed using the Hospital Anxiety and Depression Scale (HADS).^18^ The HADS exists of two components: an anxiety symptoms score (HADS-A, range 0–21) and a depression symptoms score (HADS-D, range 0–21). A score of 0–7 is considered to reflect the absence of an anxiety disorder or depression, a score of 8–10 the possibility of a disorder and ≥11 a probable disorder.^18^

### Fatigue assessment

Fatigue was assessed at first visit of the NPSLE clinic in all patients with the Dutch version of the Short-Form 36 vitality domain (SF-36 VT), which consists of four questions regarding the presence of fatigue.^19^ In addition, in patients with a first visit from 2011 onwards, the Multidimensional Fatigue Inventory (MFI)^20^ and Visual Analogue Scale (VAS) were also used to assess fatigue.

#### Fatigue and extreme fatigue

Patients with a score ≥1 standard deviation (SD) away from the mean of age-related controls of the Dutch general population on the SF-36 VT were classified as fatigued; patients ≥2 SD away were classified as extremely fatigued.^19^ In order to compare fatigue in patients with (NP)SLE with a different rheumatological disorder, the SF-36 VT of patients with rheumatoid arthritis (RA) from the early arthritis clinic of the LUMC that visited in a similar time period (2010–2018, n = 521) was obtained.^21^ Diagnosis of RA was based on the 1987 classification criteria for RA.

#### SF-36 vitality domain

Fatigue is assessed with four questions on a six-point scale ranging from “none of the time” to “all of the time”. Standardized scores range from 0 to 100 reflecting no fatigue at all.

#### Multidimensional fatigue inventory

Twenty questions are rated on a 5-point scale (“yes, that is true” to “no, that is not true”). Total scores are calculated for five subscales: general fatigue, physical fatigue, reduced activity, reduced motivation and mental fatigue. Standardized scores range from 4 to 20 per subscale. Total score was calculated by adding the subscale scores (range: 20–100), 100 reflecting the most extreme fatigue.

#### Visual analogue scale

The question “How tired were you this past week?” was rated on 11-point scale from 0 (“not tired”) to 10 (“extremely tired”).

All scores are reported as mean and SD, unless stated differently.

#### Missing data

For the SF-36 VT and MFI, item mean imputation was used if <50% of domain score was missing.^22^ There was missing data for all fatigue measurements (SF-36 VT: 8.3%, MFI: 10.5%, VAS: 11.2%), HADS (8.0%) and education level (6.2%).

### Sensitivity analyses

#### Data imputation

A sensitivity analysis was performed with multiple imputation using chained equation (for full description of the analysis, see *Supplementary File part II*) in order to provide unbiased estimates.^23^ Multiple imputation did not show differences between the results of imputed and non-imputed data, as demonstrated in *Supplemental materials part II,* Table 3. For this reason, the results of the complete case analysis are shown as main results.

**Table 3.** Association between fatigue and inflammatory neuropsychiatric symptoms, disease activity and symptoms of anxiety and depression.

	SF-36 VT		MFI total score		VAS	
	β^a^	95% CI	β	95% CI	β	95% CI
Inflammatory vs non-inflammatory (ref)	0.9	(−4.4; 6.1)	−3.7	(−6.8; −0.7)*	−1.0	(−1.7; −0.4)*
Disease activity (SLEDAI-2K)^b^	−0.1	(−1.0; 8.0)	−0.3	(−0.8; 1.5)	0.1	(−0.2; 0.5)
HADS						
Anxiety	−1.5	(−1.9; −1.1)**	0.5	(0.3; 0.8)**	0.2	(0.1; 0.2)**
Depression	−2.3	(−2.6; −2.0)**	0.9	(0.6; 1.1)**	0.2	(0.2; 0.3)**

*p < 0.05, **p < 0.001.

^a^All β’s are corrected for age, sex and education.

^b^β after back-transformation of log-transformation of SLEDAI-2K.

HADS: hospital anxiety and depression scale; MFI: multidimensional fatigue inventory; SD: standard deviation; SF-36 VT: Short Form 36 Vitality Domain; SLEDAI-2K: systemic lupus erythematosus disease activity index 2000; VAS: visual analogue scale.

#### NPSLE phenotypes

In order to assess the influence of the presence of specific NPSLE phenotypes separately, multiple sensitivity analyses were performed, as shown in *Supplemental materials part II,* Table 2.

**Table 2.** Fatigue and symptoms of anxiety/depression in patients visiting the NPSLE clinic with neuropsychiatric symptoms due to inflammation (inflammatory phenotype) and other causes (non-inflammatory phenotype).

	Inflammatory phenotype^a^(n = 72)	Non-inflammatory phenotype^b^(n = 276)
SF-36 VT	34.7 ± 20.0	34.3 ± 18.8
MFI		
General fatigue	10.8 ± 2.1	11.3 ± 2.1
Physical fatigue	11.5 ± 2.5	12.4 ± 2.2
Reduced activity	9.8 ± 3.4	10.9 ± 2.5
Reduced motivation	10.7 ± 2.6	11.3 ± 2.3
Mental fatigue	9.6 ± 3.0	10.0 ± 2.9
Total score	52.2 ± 10.6	56.0 ± 8.6
VAS	6.7 ± 2.5	7.6 ± 1.9
HADS: anxiety *(n, %)*		
No case	31 (47)	118 (45)
Possible case	12 (18)	49 (19)
* *Probable case	23 (35)	93 (36)
HADS: depression *(n, %)*		
No case	33 (52)	123 (47)
Possible case	11 (17)	57 (22)
* *Probable case	20 (31)	84 (32)

Note: Data is presented as mean ± SD, unless otherwise specified.

^a^Patients with NPSLE of inflammatory origin: inflammatory or combined phenotype NPSLE.

^b^Patients with other (NP)SLE: minor/non- NPSLE and ischemic NPSLE.

SF-36 VT was available for 93%, MFI and VAS for 89% (since 2011), HADS anxiety and HADS depression for 92%.

HADS: hospital anxiety and depression scale; MFI: multidimensional fatigue inventory; SD: standard deviation; SF-36 VT: Short Form 36 Vitality Domain; VAS: visual analogue scale.

### Statistical analyses

The association between presence of inflammatory neuropsychiatric symptoms and fatigue was studied using separate analyses for the three total scores of the fatigue assessment tools (SF-36 VT: range 0–100, MFI: range 20–100, VAS: range 0–10) in multiple regression analyses (MRA). Association between disease activity (SLEDAI-2K) and fatigue (SF-36 VT, MFI, VAS) was assessed using MRA after log-transformation for SLEDAI-2K because of non-normal distribution. MRA was also used to study the association between anxiety (HADS-A, range 0–21) and depression (HADS-D, range 0–21) and the fatigue assessment tools. All analyses were corrected for the following confounders, three important factors associated with fatigue: age, sex and education. Estimated coefficients (β) and 95% confidence intervals (CI) after correction are provided.

## Results

### Patient characteristics

In total, 371 patients visiting the NPSLE clinic were eligible, as shown in Figure 1. Questionnaires were available of 348 patients (94%), of which 72 patients had an inflammatory phenotype (21%). Patient characteristics were similar in patients with and without missing questionnaires. The mean age in inflammatory and non-inflammatory patients was 42 ± 14 and 44 ± 13 years respectively and the majority was female (88%), see Table 1.

**Table 1.** Clinical information of patients visiting the NPSLE clinic with neuropsychiatric symptoms due to inflammation (inflammatory phenotype) and other causes (non-inflammatory phenotype).

	Inflammatory phenotype^a^ (n = 72)	Non-inflammatory phenotype^b^ (n = 276)
Female	63 (88)	240 (87)
Age *(years (mean, sd))*	41.9 ± 13.8	43.7 ± 13.4
SLE duration *(years (median, range))*	1 (0–30)	5 (0–40)
ACR 1997 criteria		
Malar rash	25 (35)	112 (41)
Discoid rash	6 (8)	53 (19)
Photosensitivity	27 (38)	150 (54)
Oral ulcers	26 (36)	124 (44)
Nonerosive arthritis	51 (71)	160 (58)
Pleuritis or pericarditis	24 (33)	65 (24)
Renal disorder	21 (29)	73 (26)
Neurologic disorder	11 (15)	32 (12)
Hematologic disorder	38 (53)	132 (48)
Immunologic disorder	59 (82)	208 (75)
Positive ANA	70 (97)	269 (97)
Disease activity *(SLEDAI-2K)*		
No/mild (0-5)	28 (39)	185 (67)
Moderate (6-11)	18 (25)	70 (25)
Severe (≥12)	26 (36)	21 (8)
SDI *(median, range)*	1 (0–4)	1 (0–11)
Education level *(years)*		
Low (0-6)	3 (4)	11 (4)
Middle (6-12)	47 (65)	160 (58)
High (>12)	16 (22)	88 (32)
Unknown	5 (7)	19 (7)
Treatment		
* Anti-inflammatory*		
Corticosteroids	51 (72)	129 (47)
Azathioprine	14 (9)	40 (15)
Hydroxychloroquine	42 (58)	184 (66)
Methotrexate	3 (4)	18 (7)
Mycophenolate mofetil	31 (11)	4 (6)
Cyclophosphamide	6 (8)	1 (0)
Biologicals (rituximab, belimumab)	2 (3)	1 (0)
*Other*		
Antidepressant	12 (17)	49 (18)
Benzodiazepine	5 (22)	14 (19)

Note: Data is presented as n (%), unless otherwise specified.

^a^Patients with NPSLE of inflammatory origin: inflammatory or combined phenotype NPSLE.

^b^Patients with other (NP)SLE: minor/non-NPSLE and ischemic NPSLE.

ACR: American College of Rheumatology; NP: neuropsychiatric symptoms; SD: standard deviation; SDI: SLICC damage index; SLE: systemic lupus erythematosus; SLEDAI-2K: systemic lupus erythematosus disease activity index 2000.

Of the patients with an inflammatory phenotype, 49 patients had a pure inflammatory phenotype (68%) and 23 patients had a combined (inflammatory + ischemic) phenotype (32%). In patients with a non-inflammatory phenotype, 29 patients had an ischemic phenotype (10%) and the rest had minor/non-NPSLE (90%). All NPSLE syndromes of patients with inflammatory and ischemic NPSLE are described in Supplementary Table 1. The most common neuropsychiatric symptoms in minor/non-NPSLE patients were cognitive complaints (26%), headache (22%) and mood- or coping disorders (18%). In addition, alternative diagnoses such as infections, malignancies and side-effects of medication were present.

## Fatigue

### Prevalence

**Figure 2.**
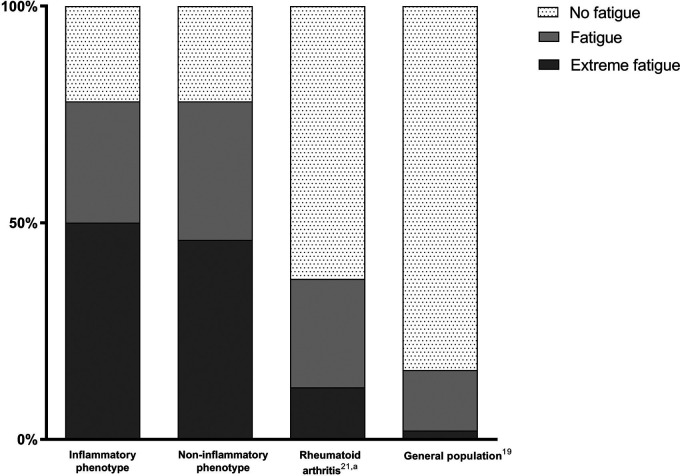
Percentage of patients with no fatigue, fatigue and extreme fatigue in SLE and neuropsychiatric symptoms of inflammatory origin (n = 72), other origin (non-inflammatory, n = 276), rheumatoid arthritis^21,a^ (n = 521) and the general Dutch population.^19^

Fatigue, defined as ≥1 SD away of the mean of age-related controls of the general population on the SF-36 VT, was present in 78% of patients with inflammatory and non-inflammatory phenotype. Extreme fatigue (≥2 SD away) was present in 50 and 46% respectively. Figure 2 illustrates the prevalence of fatigue compared with the general population and patients with early RA. Average age in the RA population was 60 ± 15 years and 62% was female.

### Inflammatory vs. non-inflammatory phenotype

The mean score of SF-36 VT was 34.7 ± 20.0 in patients with an inflammatory phenotype vs 34.3 ± 18.8 in patients with a non-inflammatory phenotype. The mean scores of the MFI domains are shown in Table 2. The SF-36 VT showed no difference in fatigue between inflammatory and non-inflammatory phenotype: β: 0.9 (95% CI −4.4; 6.1), indicating an average of 0.9 points higher on the SF-36 VT when an inflammatory phenotype is present (see Table 3). There was less fatigue in patients with an inflammatory phenotype on the MFI (β: −3.7 (95% CI: −6.8; −0.7)) and VAS (β: −1.0 (95% CI −1.7; −0.4)).

### Disease activity and fatigue

In patients with mild, moderate and severe disease activity, fatigue was present in 75%, 85% and 78% respectively. No association between disease activity and fatigue was found. The back transformed coefficients for disease activity were β: −0.1 (95% CI: −1.0; 8.0) for SF-36 VT, β: −0.3 (95% CI: −0.8; 1.5) for MFI and β: 0.1 (95% CI: −0.2; 0.5) for VAS.

### Symptoms of anxiety/depression and fatigue

In patients with no, possible and probable anxiety disorder, fatigue was present in 66%, 77% and 95% respectively. Fatigue showed a clear association between symptoms of anxiety (measured by the HADS-A): SF-36 VT: β: −1.5 (95% CI: −1.9; −1.1), MFI: β: 0.5 (95% CI: 0.3; 0.8) and VAS: β: 0.2 (95% CI: 0.1; 0.2). In patients with no, possible and probable depression, fatigue was present in 61%, 90% and 96% respectively. A strong association between depressive symptoms (measured by the HADS-D) and fatigue was present: SF-36 VT: β: −2.3 (95% CI: −2.6; −2.0), MFI: β: 0.9 (95% CI: 0.6; 1.1) and VAS: β: 0.2 (95% CI: 0.2; 0.3).

### Sensitivity analyses

Different sensitivity analyses yielded similar results as the main analyses, as shown in Supplementary Tables 2 and 3.

## Discussion

In this study, we analyzed fatigue in patients with SLE presenting with different neuropsychiatric manifestations and demonstrated that nearly 80% of all patients were fatigued. We found no association between inflammation and fatigue, but symptoms of anxiety and depression associated strongly with fatigue. This suggests that a therapeutic strategy directed at affective symptoms might be more effective than a strategy based on immunomodulation in order to treat fatigue in patients with SLE and neuropsychiatric symptoms.

Previous research has estimated the prevalence of fatigue in patients with SLE between 67–90%.^1^ In clinical practice, many different tools are used to assess fatigue and different definitions of fatigue are used throughout studies in patients with SLE, complicating direct comparison across studies.^24,25^ One study used a cut-off score of 35 of the SF-36 VT to define severe fatigue and showed a prevalence of 52% in SLE patients.^26^ Severe fatigue was present in 57% of patients with inflammatory and non-inflammatory neuropsychiatric symptoms of our cohort using the same cut-off. This suggests that, although we have a selection of patients with SLE and neuropsychiatric symptoms, fatigue prevalence appears to be similar to what has been described in non-NPSLE patients on the SF-36 VT. The MFI assesses fatigue in more detail than the SF-36 VT and provides insight in different aspects of fatigue (general fatigue, physical fatigue, reduced activity, reduced motivation and mental fatigue).^20^ It has also been suggested to be the most sensitive tool to detect differences in fatigue levels in SLE patients,^27^ but only a few studies have used the MFI in patients with SLE.^8,26–28^ One small study in 21 women with SLE showed less fatigue on all MFI domains compared to ours, which might be explained by a slightly younger average age of this study population and lower scores on the HADS.^28^ Our results are in line with two other studies, which reported similar scores on the five subdomains (range: 8–15).^8,26^ Interestingly, although these studies did not specifically include patients with SLE and neuropsychiatric symptoms, the domain ‘mental fatigue’ was similar in severity. In all SLE patients, including ours, physical and general fatigue appears to be the most prominent.^8,26,28^

Based on previous research, the relationship between inflammation and fatigue in patients with SLE remains unclear. This is the first study to compare fatigue in SLE patients with inflammatory vs non-inflammatory neuropsychiatric symptoms, and inflammation of the nervous system did not increase the presence or severity of fatigue. Disease activity as a measure of inflammation has shown contradictory results: some reports have suggested an association, whereas the majority, including ours, do not.^2^ It has been suggested that the association might differ depending on the method used to study disease activity (patient reported vs e.g. SLEDAI-2K).^29^ However, other measurements of inflammation, such as proinflammatory cytokines, are consistently associated with fatigue in both SLE and other autoimmune diseases.^3^ There are several underlying mechanisms which are thought to contribute to fatigue in autoimmune diseases, mainly through brain inflammation. In patients with autoimmune diseases, increased stress levels are reported.^30^ Stress leads to a response of the hypothalamic-pituitary-adrenal axis, including the release of corticotropin-releasing-hormone.^3^ As a result of chronic stress, reduced glucocorticoid sensitivity can occur, which promotes local proinflammatory signaling, resulting in fatigue.^3^ In addition, cytokines can enter the brain through different mechanisms, where they can affect neurotransmitters involved in fatigue.^31^ Although these processes can result in fatigue in all autoimmune diseases, our clinical experience is that the prevalence and burden of fatigue seems to be much higher in SLE patients than in e.g. other rheumatic autoimmune diseases. In this study, we indeed demonstrated that patients with (NP)SLE showed more fatigue than patients with early RA: 78% vs 37% (Figure 2). Although the number of patients with fatigue is still clearly increased in early RA compared to the general population, the difference between early RA and SLE is striking.

Anxiety and major depression are common in patients with SLE, with an estimated prevalence of 37% and 24% respectively based on clinical interview.^32,33^ Symptoms of depression and anxiety (as measured by the HADS) have been reported to be higher in patients with SLE than RA, which serves as a possible explanation for the increased prevalence of fatigue in patients with SLE.^34^ Many studies have found an association between fatigue and symptoms of depression and anxiety in patients with SLE.^2,35,36^ Using the HADS, we also found a high prevalence of symptoms of anxiety and depression and confirmed its association with fatigue. One-third of all patients had ≥11 points on the anxiety and depression subscales (range 0–21), indicating a probable disorder. This number is much higher than the number of patients with an actual (DSM-V) diagnosis established during the psychiatric assessment, which was present in 5% (anxiety) and 23% (depression) in our population. Although a high HADS score might not always reflect an accurate clinical diagnosis of anxiety or depression, it does emphasize the common occurrence of these symptoms. As we have shown that these symptoms already associate strongly with fatigue, addressing these symptoms might reduce fatigue and increase quality of life.

Fatigue is often insufficiently addressed by both physicians and patients with SLE,^37^ despite its known impact on daily life, interference with work and ability to reduce quality of life.^38^ Health care professionals should actively ask about fatigue, resources and coping strategies and provide information regarding the management of fatigue.^37^ Management strategies such as psychosocial intervention (e.g. behavioral therapy) and exercise have already shown to reduce fatigue in patients with SLE.^39,40^ These strategies reduced depressive symptoms and improved cognition, both common neuropsychiatric symptoms in SLE patients and potential contributors to fatigue.^41^ Therefore, we believe that these strategies should be actively discussed with all SLE patients presenting with neuropsychiatric symptoms and fatigue.

An important strength of our study is the well-defined population of patients with SLE and neuropsychiatric symptoms. In addition, fatigue was measured using different tools and consistently showed no influence of inflammation on fatigue. There are, however, also several limitations to our study. Firstly, as this is an observational study, results should be interpreted with caution and further studies are necessary to evaluate the influence of the suggested different therapeutic strategies on fatigue. In addition, other aspects that influence fatigue, such as cognitive deficits and sleep disturbances were not studied in detail and could also be potential targets for intervention. Lastly, as the NPSLE clinic is a tertiary referral center, this study population entails more complex cases than regular (NP)SLE patients. However, despite this selection of cases, we have demonstrated that our results are similar to other (NP)SLE populations.

In conclusion, we demonstrate that patients with SLE and neuropsychiatric symptoms are extremely fatigued, independent of underlying etiology (inflammatory vs. non-inflammatory) of these symptoms and disease activity. As fatigue is strongly associated with anxiety and/or depressive symptoms, targeting mood (disorders) may be an appropriate treatment strategy.

## Supplemental Material

sj-pdf-1-lup-10.1177_09612033211005014 - Supplemental material for Fatigue in patients with systemic lupus erythematosus and neuropsychiatric symptoms is associated with anxiety and depression rather than inflammatory disease activity Click here for additional data file. Supplemental material, sj-pdf-1-lup-10.1177_09612033211005014 for Fatigue in patients with systemic lupus erythematosus and neuropsychiatric symptoms is associated with anxiety and depression rather than inflammatory disease activity by Rory C Monahan, Liesbeth JJ Beaart-van de Voorde, Jeroen Eikenboom, Rolf Fronczek, Margreet Kloppenburg, Huub AM Middelkoop, Gisela M Terwindt, Nic JA van der Wee, Tom WJ Huizinga and Gerda M Steup-Beekman in Lupus

## References

[1] Cleanthous S Tyagi M Isenberg Dand Newman SP. What do we know about self-reported fatigue in systemic lupus erythematosus? Lupus 2012; 21: 465–476. 22345120 10.1177/0961203312436863

[2] Ahn GE Ramsey-Goldman R. Fatigue in systemic lupus erythematosus. Int J Clin Rheumtol 2012; 7: 217–227. 22737181 10.2217/IJR.12.4 PMC3380630

[3] Zielinski MR Systrom DM Rose NR. Fatigue, sleep, and autoimmune and related disorders. Front Immunol 2019; 10: 1827. 31447842 10.3389/fimmu.2019.01827 PMC6691096

[4] Merrill JT Burgos-Vargas R Westhovens R, et al. The efficacy and safety of abatacept in patients with non-life-threatening manifestations of systemic lupus erythematosus: results of a twelve-month, multicenter, exploratory, phase IIb, randomized, double-blind, placebo-controlled trial. Arthritis Rheum 2010; 62: 3077–3087. 20533545 10.1002/art.27601

[5] Strand V Levy RA Cervera R, et al. Improvements in health-related quality of life with belimumab, a B-lymphocyte stimulator-specific inhibitor, in patients with autoantibody-positive systemic lupus erythematosus from the randomised controlled BLISS trials. Ann Rheum Dis 2014; 73: 838–844. 23524886 10.1136/annrheumdis-2012-202865 PMC3995218

[6] Demyttenaere K De Fruyt J Stahl SM. The many faces of fatigue in major depressive disorder. Int J Neuropsychopharmacol 2005; 8: 93–105. 15482632 10.1017/S1461145704004729

[7] Jump RL Robinson ME Armstrong AE, Barnes EV, Kilbourn KM and Richards HB. Fatigue in systemic lupus erythematosus: contributions of disease activity, pain, depression, and perceived social support. J Rheumatol 2005; 32: 1699–1705. 16142863

[8] Da Costa D Dritsa M Bernatsky S, et al. Dimensions of fatigue in systemic lupus erythematosus: relationship to disease status and behavioral and psychosocial factors. J Rheumatol 2006; 33: 1282–1288. 16758508

[9] Kozora E Ellison MC West S. Depression, fatigue, and pain in systemic lupus erythematosus (SLE): relationship to the American College of Rheumatology SLE neuropsychological battery. Arthritis Rheum 2006; 55: 628–635. 16874786 10.1002/art.22101

[10] Hanly JG McCurdy G Fougere L and Douglas J-A. Neuropsychiatric events in systemic lupus erythematosus: attribution and clinical significance. J Rheumatol 2004; 31: 2156–2162. 15517627

[11] Hanly JG Kozora E Beyea SD and Birnbaum J. Nervous system disease in systemic lupus erythematosus: current status and future directions. Arthritis Rheumatol 2019; 71: 33–42. 29927108 10.1002/art.40591

[12] Gladman DD Ibanez D Urowitz MB. Systemic lupus erythematosus disease activity index 2000. J Rheumatol 2002; 29: 288–291. 11838846

[13] Morris G Berk M Galecki P, Walder K and Maes M. The Neuro-immune pathophysiology of central and peripheral fatigue in systemic immune-inflammatory and neuro-immune diseases. Mol Neurobiol 2016; 53: 1195–1219. 25598355 10.1007/s12035-015-9090-9

[14] Morris G Berk M Walder K and Maes M. Central pathways causing fatigue in neuro-inflammatory and autoimmune illnesses. BMC Med 2015; 13: 28. 25856766 10.1186/s12916-014-0259-2 PMC4320458

[15] Zirkzee EJ Steup-Beekman GM van der Mast RC, et al. Prospective study of clinical phenotypes in neuropsychiatric systemic lupus erythematosus; multidisciplinary approach to diagnosis and therapy. J Rheumatol 2012; 39: 2118–2126. 22984275 10.3899/jrheum.120545

[16] Monahan RC Fronczek R Eikenboom J, et al. Mortality in patients with systemic lupus erythematosus and neuropsychiatric involvement: a retrospective analysis from a tertiary referral center in The Netherlands. Lupus 2020; 29: 1892–1901. 33079617 10.1177/0961203320963815 PMC7684795

[17] The American College Of rheumatology nomenclature and case definitions for neuropsychiatric lupus syndromes. Arthritis Rheum 1999; 42: 599–608. 10211873 10.1002/1529-0131(199904)42:4<599::AID-ANR2>3.0.CO;2-F

[18] Zigmond AS Snaith RP. The hospital anxiety and depression scale. Acta Psychiatr Scand 1983; 67: 361–370. 6880820 10.1111/j.1600-0447.1983.tb09716.x

[19] Aaronson NK Muller M Cohen PDA, et al. Translation, validation, and norming of the Dutch language version of the SF-36 health survey in community and chronic disease populations. J Clin Epidemiol 1998; 51: 1055–1068. 9817123 10.1016/s0895-4356(98)00097-3

[20] Smets EMA Garssen B Bonke B and De Haes JC. The multidimensional fatigue inventory (MFI) psychometric qualities of an instrument to assess fatigue. J Psychosom Res 1995; 39: 315–325. 7636775 10.1016/0022-3999(94)00125-o

[21] van Aken J van Bilsen JH Allaart CF, Huizinga TW and Breedveld FC. The Leiden early arthritis clinic. Clin Exp Rheumatol 2003; 21: S100–105. 14969059

[22] Ware JE Kosinski M Keller SD. SF-36 physical and mental health summary scales: a user's manual. 5th ed. Boston, MA: Health Assessment Lab, New England Medical Center, 1994.

[23] Azur MJ Stuart EA Frangakis C and Leaf PJ. Multiple imputation by chained equations: what is it and how does it work? Int J Methods Psychiatr Res 2011; 20: 40–49. 21499542 10.1002/mpr.329 PMC3074241

[24] Ad Hoc Committee on Systemic Lupus Erythematosus Response Criteria for F. Measurement of fatigue in systemic lupus erythematosus: a systematic review. Arthritis Rheum 2007; 57: 1348–1357. 18050225 10.1002/art.23113

[25] Barbacki A Petri M Avina-Zubieta AAlarcon GS and Bernatsky S. Fatigue measurements in systemic lupus erythematosus. J Rheumatol 2019; 46: 1470–1477. 30709953 10.3899/jrheum.180831

[26] Overman CL Kool MB Da Silva JA and Greenen R. The prevalence of severe fatigue in rheumatic diseases: an international study. Clin Rheumatol 2016; 35: 409–415. 26272057 10.1007/s10067-015-3035-6 PMC4752960

[27] Goligher EC Pouchot J Brant R, et al. Minimal clinically important difference for 7 measures of fatigue in patients with systemic lupus erythematosus. J Rheumatol 2008; 35: 635–642. 18322987

[28] Moraleda V Prados G Martinez MPSánchez AI, Sabio JM and Prados G. Sleep quality, clinical and psychological manifestations in women with systemic lupus erythematosus. Int J Rheum Dis 2017; 20: 1541–1550. 28425178 10.1111/1756-185X.13081

[29] Kernder A Elefante E Chehab G, Tani C, Mosca M and Schneider M. The patient's perspective: are quality of life and disease burden a possible treatment target in systemic lupus erythematosus? Rheumatology (Oxford) 2020; 59: v63–v68. 33280017 10.1093/rheumatology/keaa427 PMC7719037

[30] Karagkouni A Alevizos M Theoharides TC. Effect of stress on brain inflammation and multiple sclerosis. Autoimmun Rev 2013; 12: 947–953. 23537508 10.1016/j.autrev.2013.02.006

[31] Korte SM Straub RH. Fatigue in inflammatory rheumatic disorders: pathophysiological mechanisms. Rheumatology (Oxford) 2019; 58: v35–v50. 31682277 10.1093/rheumatology/kez413 PMC6827268

[32] Palagini L Mosca M Tani CGemignana A, Mauri M and Bombardieri S. Depression and systemic lupus erythematosus: a systematic review. Lupus 2013; 22: 409–416. 23427220 10.1177/0961203313477227

[33] Zhang L Fu T Yin RZhang Q and Shen B. Prevalence of depression and anxiety in systemic lupus erythematosus: a systematic review and meta-analysis. BMC Psychiatry 2017; 17: 70. 28196529 10.1186/s12888-017-1234-1 PMC5310017

[34] Figueiredo-Braga M Cornaby C Cortez A, et al. Depression and anxiety in systemic lupus erythematosus: the crosstalk between immunological, clinical, and psychosocial factors. Medicine (Baltimore) 2018; 97: e11376. 29995777 10.1097/MD.0000000000011376 PMC6076116

[35] Moldovan I Cooray D Carr F, et al. Pain and depression predict self-reported fatigue/energy in lupus. Lupus 2013; 22: 684–689. 23660302 10.1177/0961203313486948

[36] Azizoddin DR Gandhi N Weinberg S, Sengupta M, Nicassio PM, Jolly M. Fatigue in systemic lupus: the role of disease activity and its correlates. Lupus 2019; 28: 163–173. 30580659 10.1177/0961203318817826

[37] Kier AO Midtgaard J Hougaard KS, et al. How do women with lupus manage fatigue? A focus group study. Clin Rheumatol 2016; 35: 1957–1965. 27225245 10.1007/s10067-016-3307-9

[38] Palagini L Tani C Mauri M, et al. Sleep disorders and systemic lupus erythematosus. Lupus 2014; 23: 115–123. 24421291 10.1177/0961203313518623

[39] Yuen HK Cunningham MA. Optimal management of fatigue in patients with systemic lupus erythematosus: a systematic review. Ther Clin Risk Manag 2014; 10: 775–786. 25328393 10.2147/TCRM.S56063 PMC4199565

[40] Mahieu MA Ahn GE Chmiel JS, et al. Fatigue, patient reported outcomes, and objective measurement of physical activity in systemic lupus erythematosus. Lupus 2016; 25: 1190–1199. 26869353 10.1177/0961203316631632 PMC4980272

[41] Swenson S Blum K McLaughlin TGold MS and Thanos PK. The therapeutic potential of exercise for neuropsychiatric diseases: a review. J Neurol Sci 2020; 412: 116763. 32305746 10.1016/j.jns.2020.116763

