# Supplementary material for: Fatigue in patients with systemic lupus erythematosus and neuropsychiatric symptoms is associated with anxiety and depression rather than inflammatory disease activity
Source: Lupus. 2021 Mar 28;30(7):1124–32. doi: 10.1177/09612033211005014 (PMC8120630; doi:10.1177/09612033211005014)
Supplement: sj-pdf-1-lup-10.1177_09612033211005014 - Supplemental material for Fatigue in patients with systemic lupus erythematosus and neuropsychiatric symptoms is associated with anxiety and depression rather than inflammatory disease activity [file sj-pdf-1-lup-10.1177_09612033211005014.pdf]

Supplementary file part I

Supplementary Table 1 NPSLE syndromes according to 1999 ACR criteria of patients with SLE and neuropsychiatric symptoms attributed to SLE (NPSLE)

	**Inflammatory phenotype**[a] (n = 72)	**Ischemic phenotype** (n = 29)
**NPSLE syndrome**		
Aseptic meningitis	1 (1)	0 (0)
Cerebrovascular disease	20 (28)	27 (93)
Demyelinating syndrome	0 (0)	0 (0)
Headache	7 (10)	1 (3)
Movement disorder (chorea)	3 (4)	0 (0)
Myelopathy	7 (10)	0 (0)
Seizure disorders	6 (8)	3 (10)
Acute confusional state	7 (10)	0 (0)
Anxiety disorder	1 (1)	0 (0)
Cognitive dysfunction	33 (46)	2 (7)
Mood disorder	12 (17)	0 (0)
Psychosis	7 (10)	0 (0)
AIDP[b]	0 (0)	0 (0)
Autonomic disorder	0 (0)	0 (0)
Mononeuropathy	0 (0)	0 (0)
Myasthenia gravis	0 (0)	0 (0)
Neuropathy, cranial	6 (8)	0 (0)
Plexopathy	0 (0)	0 (0)
Polyneuropathy	5 (7)	0 (0)
Other[c]	19 (26)	0 (0)

Data is presented as n (%).

[a] Patients with NPSLE of inflammatory origin (n = 72): inflammatory or combined NPSLE phenotype.

[b] Acute inflammatory demyelinating polyneuropathy.

[c] Other NPSLE symptoms: cerebral vasculitis (n = 8), organic brain syndrome (n = 3), lethargy (n = 1) visual disturbance other than optic neuritis (n =1), apraxia (n =1), walking disorder (n =2), motor disorder left arm (n =1), paresis left arm and dysarthria (n = 1), increased intracranial pressure (n =1).

Supplementary file part II

Sensitivity analyses multiple imputation

A sensitivity analyses was performed with multiple imputation using chained equation (MICE) in STATA version 14.1. Sex, age, ACR criteria, disease duration, disease activity score (SLEDAI-2K), damage index (SDI), NPSLE and NPSLE phenotype and medication use were used for imputation (n = 50) using predictive mean matching.

Supplementary Table 2 Result of sensitivity analysis using multiple imputation for the analysis of fatigue in patients with neuropsychiatric symptoms due to inflammation (inflammatory phenotype) and other causes (non-inflammatory phenotype)

	Inflammatory phenotype[a] Main analysis	**Inflammatory phenotype[a] n = 81**	Non-inflammatory phenotype[b] Main analysis	**Non-inflammatory phenotype[b] n = 290**
**Fatigue** (%)	77.9	78.5	78.3	77.9
**Extreme fatigue** (%)	50.0	49.8	46.3	45.8
**SF-36 VT** *(mean, SE)*	34.7	34.3 ± 2.3	34.5	34.3 ± 1.1
**MFI** *(mean, SE)*				
General Fatigue	10.8	10.8 ± 0.3	11.3	11.3 ± 0.2
Physical Fatigue	11.5	11.6 ± 0.3	12.4	12.4 ± 0.2
Reduced Activity	9.8	9.8 ± 0.5	10.9	10.8 ± 0.2
Reduced Motivation	10.7	10.8 ± 0.4	11.3	11.3 ± 0.2
Mental Fatigue	9.6	9.6 ± 0.4	10.0	10.0 ± 0.2
Total score	52.2	52.6 ± 1.4	56.0	55.7 ± 0.6
**VAS** *(mean, SE)*	6.7	6.8 ± 0.3	7.6	7.6 ± 0.1

[a] Patients with NPSLE of inflammatory origin: inflammatory or combined phenotype NPSLE.

[b] Patients with non-inflammatory (NP)SLE: minor/non-NPSLE and ischemic NPSLE.

MFI = multidimensional fatigue inventor, SE = standard error, SF-36 VT = Short-Form 36 Vitality Domain, VAS = visual analogue scale.

Supplementary Table 3 Sensitivity analyses studying the influence of the presence of neuropsychiatric symptoms due to inflammation (inflammatory phenotype) and other causes (non-inflammatory phenotype)

	**SF-36 VT**		**MFI total score**		**VAS**	
	β*	95% CI	β	95% CI	β	95% CI
Inflammatory vs non-inflammatory (ref) (main result)	0.9	(-4.4; 6.1)	-3.7	(-6.8; -0.7)	-1.0	(-1.7; -0.4)
Inflammatory vs non-inflammatory (ref) after multiple imputation	0.1	(-5.0; 4.8)	-3.0	(-5.8; -0.3)	-0.9	(-1.5; -0.3)
Inflammatory vs minor/non-NPSLE (ref) (excluding ischemic NPSLE)	1.4	(-3.9; 6.6)	-4.0	(-7.1; -0.8)	-1.1	(-1.7; -0.4)
Inflammatory vs non-inflammatory (ref) (excluding combined phenotype)	2.0	(-4.1; 8.0)	-3.2	(-6.8; 0.5)	-0.9	(-1.6; -0.1)
Inflammatory vs minor/non-NPSLE (ref) (excluding ischemic + combined)	2.5	(-3.5; 8.5)	-3.4	(-7.1; 0.3)	-0.9	(-1.7; -0.2)
NPSLE vs minor/non-NPSLE (ref)	2.3	(-2.4; 7.0)	-3.4	(-6.2; -0.7)	-0.9	(-1.5; -0.3)

* All β's are corrected for age, sex and education.

MFI = multidimensional fatigue inventory, SF-36 VT = Short-Form 36 Vitality Domain, VAS = visual analogue scale.
